# Behçet’s disease: successful aortic root reconstruction in severely dilated aortoventricular junction after aortic valve replacement with novel surgical method – case report

**DOI:** 10.1186/s13019-021-01467-1

**Published:** 2021-04-15

**Authors:** Miklós Pólos, Ádám Koppányi, Kálmán Benke, László Daróczi, Attila Oláh, Krisztina Heltai, Emese Kiss, Attila Fintha, Beáta Nagy, István Hartyánszky, Bálint Lakatos, Attila Kovács, Béla Merkely, Zoltán Szabolcs

**Affiliations:** 1 Semmelweis University, Heart and Vascular Center, Varosmajor 68, Budapest, H-1122 Hungary; 2 National Institute of Rheumatology and Physiotherapy, Budapest, Hungary; 3 First Department of Pathology and Experimental Cancer Research, Semmelweis University, Budapest, Hungary

**Keywords:** Behçet’s disease, Aortic root reconstruction, Modified Bentall procedure, Case report

## Abstract

**Background:**

Behçet’s disease is an auto-inflammatory disorder categorized as a primer systemic vasculitis of unknown aetiology. Genetic factors, infectious agents and the irregularity of T-cell homeostasis are presumed to be responsible for the emergence of Behçet’s disease. Characteristic symptoms are multisystemic. Although cardiovascular involvement is rare, it should be noted due to the difficulty of surgical treatment options.

**Case presentation:**

Our 44-year-old male patient underwent aortic valve replacement due to aortic regurgitation. At the 15-month follow-up, echocardiography showed detachment of the prosthetic valve and in the aortic root, multiple pseudo-aneurysms were identified. We performed an aortic root reconstruction with a Bentall procedure using a special „skirted” conduit to reduce strain in the suture line between the conduit and the extremely dilated left ventricular outflow tract.

**Conclusions:**

The surgical treatment of cardiovascular manifestations of Behçet’s disease remains challenging. This new technique may be beneficial in well-selected cases where the annulus of the aorta is extremely dilated or annular tissue disorder is present.

## Background

Behçet’s disease (BD) belongs to primary systemic vasculitides without vessel preference according to the Chapel Hill Consensus Conference classification [1]. The most common and typical symptoms include painful oral and genital ulcers, skin rashes, pathergy, inflammatory arthritis, and eye inflammation that may lead to blindness. Cardiovascular manifestations occur in approximately 30% of BD patients. Histopathologically, the vasculitis is characterized by infiltration with neutrophil granulocytes, endothelial cell activation with consequent appearance of anti-endothelial antibodies, fibrinoid necrosis, destruction of media, and periadventitial fibrosis [2]. Cardiac involvement includes valvular diseases, pericarditis, myocarditis, and endocarditis [3]. As cardiac and/or large vessel manifestation, aortic regurgitation can be present in 1,2-2,3% of BD patients [4].

Aetiology has not fully been cleared yet. Current knowledge presumes that genetic and environmental factors are both involved. Approximately 60% of Behçet’s patients carry human leukocyte antigen (HLA)-B51 allele, but other genetic polymorphisms have also been identified as aetiological factors [5, 6].

Clinical manifestations are heterogeneous. Recurrent oral aphthous lesions appear more than 3 times in a year. This is the major criterion for the classification of the disease according to the International Study Group for Behçet’s Disease [7]. Minor criteria are 1. genital ulceration, 2. uveitis, 3. skin rashes (erythema nodosum, pustulosis, acne), and 4. pathergy. Diagnosis is based on characteristic symptoms, and the exclusion of other diseases, as no specific biochemical markers have been identified until now [6].

Treatment remains challenging. As first line of treatment, corticosteroids are used to induce remission. Thalidomide and Dapsone have also been used as immune-modulators. Immunosuppressants such as Azathioprine, Cyclosporine A, Methotrexate, and Cyclophosphamide have steroid-sparing effect and can be chosen in severe cases. Biological or targeted synthetic therapies also have a reasonable therapeutic role [5].

In patients suffering from BD, the annulus of the aorta and the aortic wall are fragile due to inflammation in the endothelial tissue [8]. Patients with BD who underwent aortic valve replacement have a higher risk for recurrent postoperative valve detachment than patients with normal annular and aortic wall tissue. The incidence of prosthetic valve dehiscence is 40–78% in BD patients. Prosthetic valve dislocation can lead to acute cardiogenic shock through severe aortic regurgitation (AR). Dehiscence along the suture lines or rupture of the pseudo-aneurysm may cause pericardial tamponade and can lead to fatal outcomes [9]. To prevent postoperative complications and to reduce mortality after aortic valve replacement, several surgical methods have been reported, such as the use of transmural buttress sutures to fix prosthetic valves at the annulus, the use of a valved-conduit or homograft, as well as the Bentall procedure and modified Bentall procedure [10]. However, there is no definite surgical approach for AR in BD patients. As a laboratory marker, only the role of low C-reactive protein (CRP) concentration is mentioned to correlate with an uneventful postoperative course [11].

## Case presentation

### Patient information

Our patient is a 44-year-old male. In his medical history urticaria-like skin eruption was present persistently since 2008. Inflammatory bowel disease was confirmed a year later, when histopathology was similar to findings in ulcerative colitis. In 2013 major skin lesions appeared and were diagnosed as pyoderma gangrenosum. Methylprednisolone and azathioprine were administered but had to be changed to methotrexate due to inefficacy in 2015.

In 2018 echocardiography was performed due to progressive worsening of shortness of breath. Severe aortic regurgitation was found, with moderately decreased left ventricular systolic function (ejection fraction 50%) and massive enlargement of the left ventricle (ESD 6.6 cm, EDD 8.2 cm). Right ventricular dimensions and function were preserved. Other valves were intact. CT angiography ruled out atherosclerotic disease of the aortic root and of the coronary arteries. Preoperative CRP concentration was mildly elevated (9 mg/l).

In May 2018 the patient underwent regular aortic valve replacement (AVR, LivaNova Bicarbon 23 mm; LivaNova PLC, London, UK). The postoperative period was uneventful. Postoperative echocardiography revealed decreased left ventricular systolic function (ejection fraction 30%) along with improving ventricular diameters. CRP concentrations were appropriate to the present postoperative status (69 mg/l on postoperative day four). Three months following the surgery, echocardiography revealed improvement of left ventricular function (ejection fraction 42%) and normally functioning prosthetic valve. CRP concentration was 19 mg/l.

### Clinical findings

In September 2019 a follow-up echocardiography was performed which confirmed severe paravalvular regurgitation. The appearance of the aortic root was highly suggestive of prosthetic valve dehiscence.

### Diagnostic assessment and timeline

**Fig. 1:**
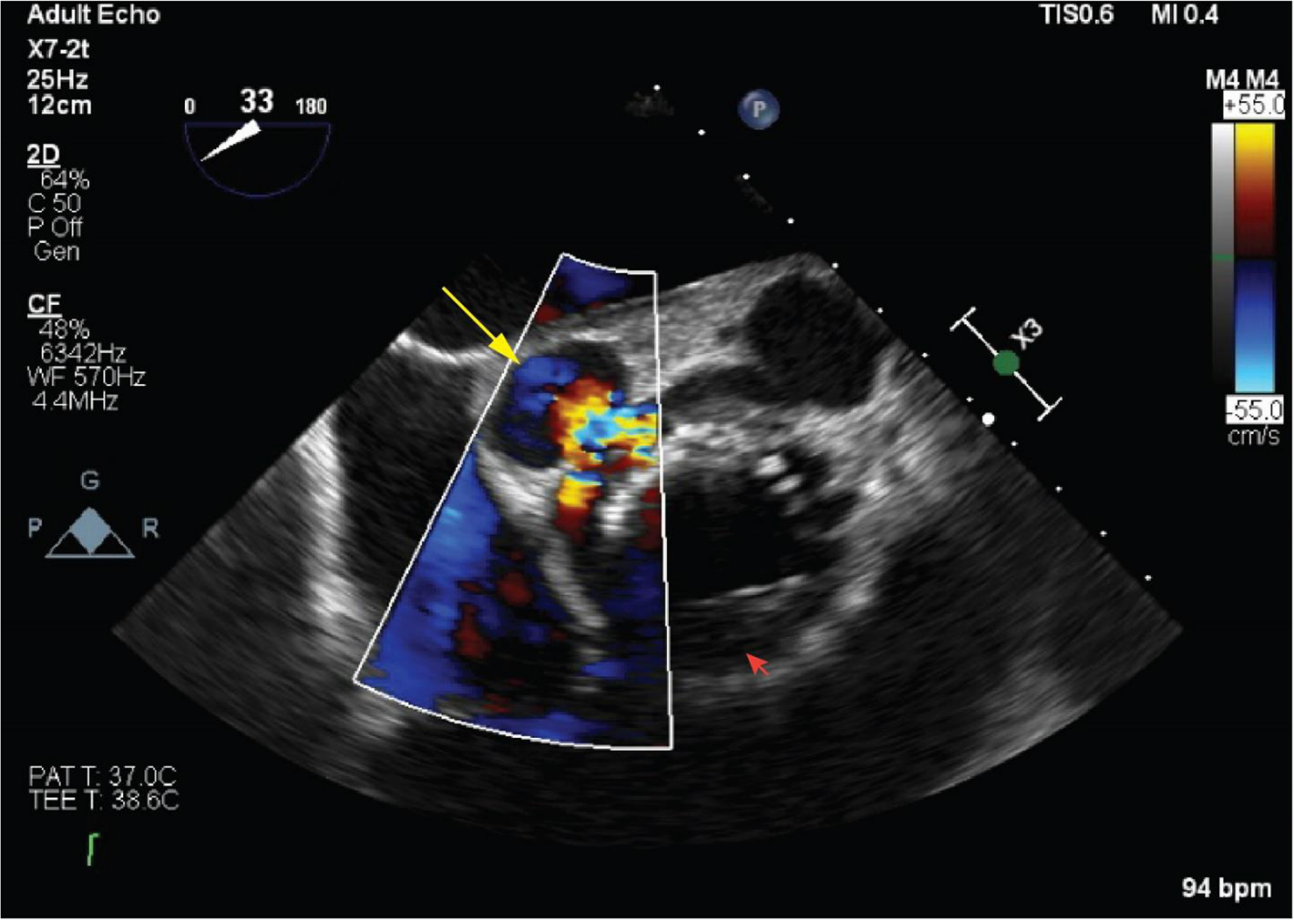
Preoperative echocardiography: Preoperative imaging showing severe aortic regurgitation and pseudo-aneurysms originating from the aortic annulus (yellow arrow). A shallow membrane is protruding from the aortic annulus, fixing the prosthetic aortic valve (red arrow)

**Fig. 2:**
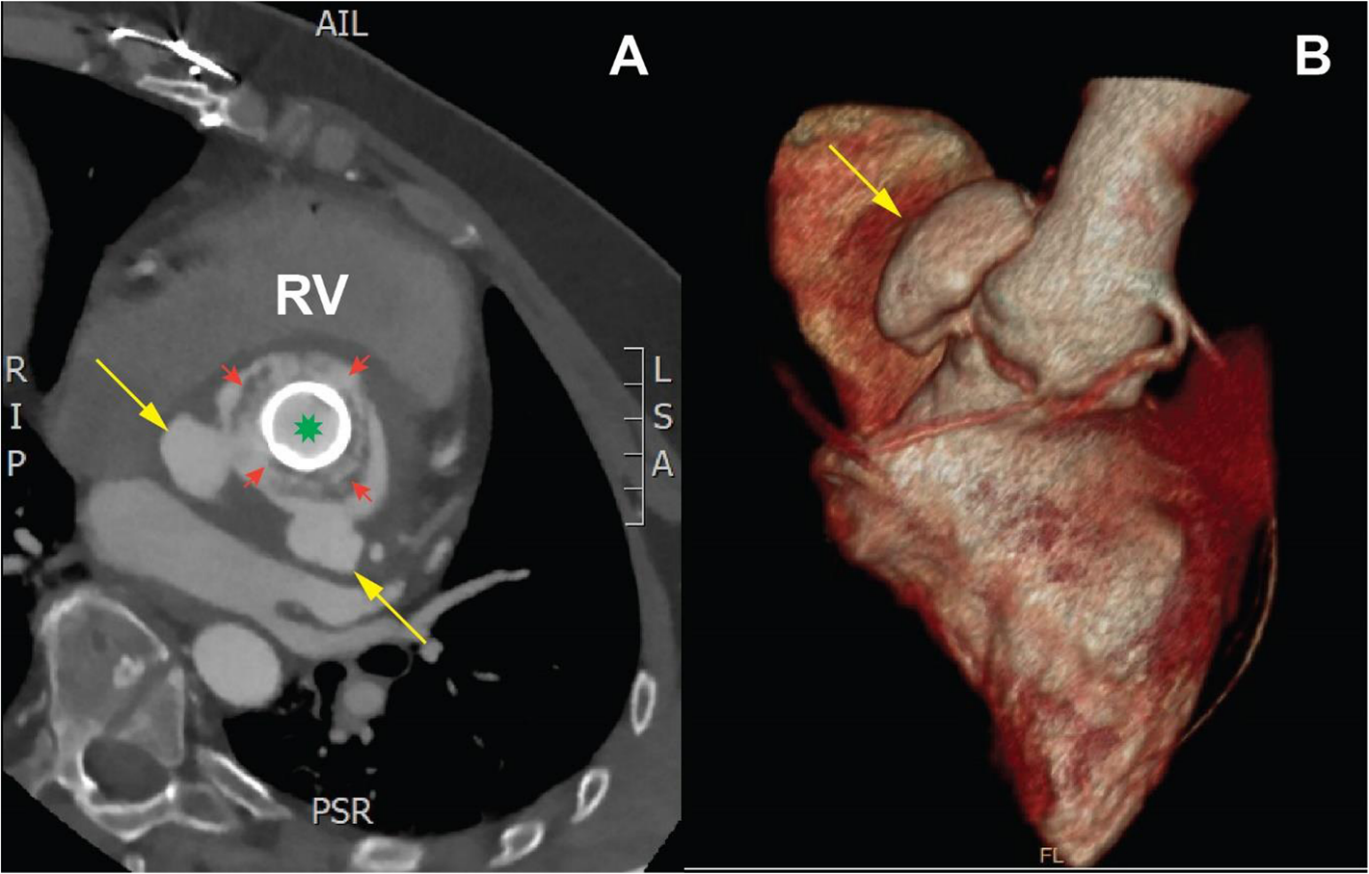
Preoperative chest CT-angiography: **a:** Two pseudo-aneurysms originate from the aortic annulus, specifically from the non-coronary sinus and from behind the left main coronary branch (yellow arrows). A shallow membrane (red arrows) is fixing the dehiscenced prosthetic aortic valve (green mark) (**b)**: 3D reconstruction of chest CT-angiography showing the pseudo-aneurysm arising from the non-coronary sinus (yellow arrow)

Transoesophageal echocardiography revealed high mobility of the prosthetic valve along with localized dissection of the outflow tract resulting in a „Mickey Mouse” like appearance of the aortic root. (Fig. 1.) Blood tests did not show elevated inflammatory markers, while blood cultures were also negative. Chest CT angiography scan revealed two pseudo-aneurysms originating from the non-coronary sinus of the aorta and from behind the left main coronary branch. (Fig. 2.)

### Therapeutic intervention

As the result of a multidisciplinary consultation, we decided to perform aortic root reconstruction. Due to the enlargement of the left ventricular outflow tract to 43 mm, presumably, the use of a regular Bentall conduit would not have been favourable. The use of a rigid suture ring could have led to an exceedingly big strain on the suture line between the extremely dilated annulus and the conduit. To reduce strain in the suture line, we created a „skirted” conduit by implanting a 29 mm mechanical prosthetic valve (SJM 29 mm; St Jude Medical, St Paul, USA) into a 36 mm diameter vascular prosthesis (Vascutek Ltd., Scotland, UK), leaving a 2 cm free end as the „skirt” of the graft.

**Fig. 3:**
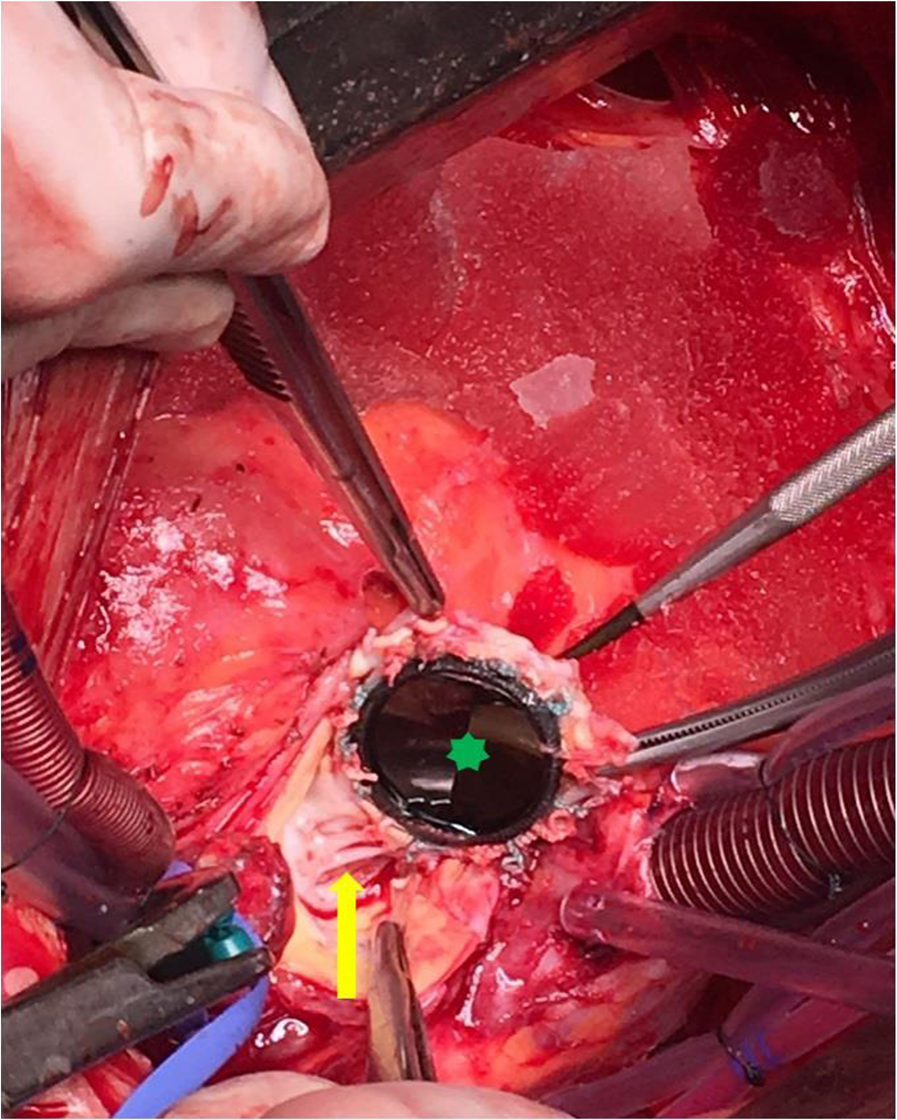
Intraoperative picture of the prostethic-valve dehiscence: Intraoperative findings show the dehiscence of the prosthetic aortic valve (green mark). A shallow membrane is fixing the prosthetic valve, protruding from the aortic annulus (yellow arrow)

**Fig. 4:**
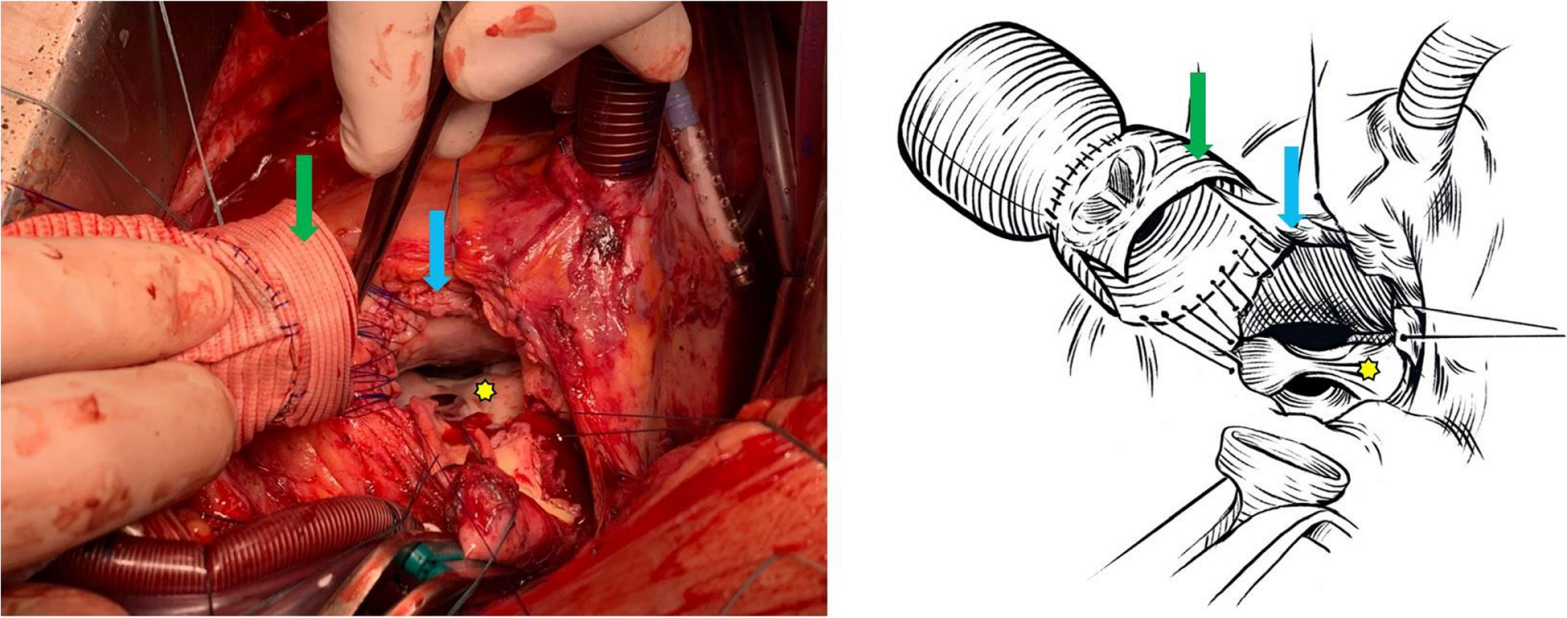
Intraoperative and schematic picture of the “skirted” graft: Intaoperative status shows the “skirted” graft (green arrow) sutured directly to the left ventricular outflow tract (blue arrow). The anterior leaflet of the mitral valve is marked with yellow

**Fig. 5:**
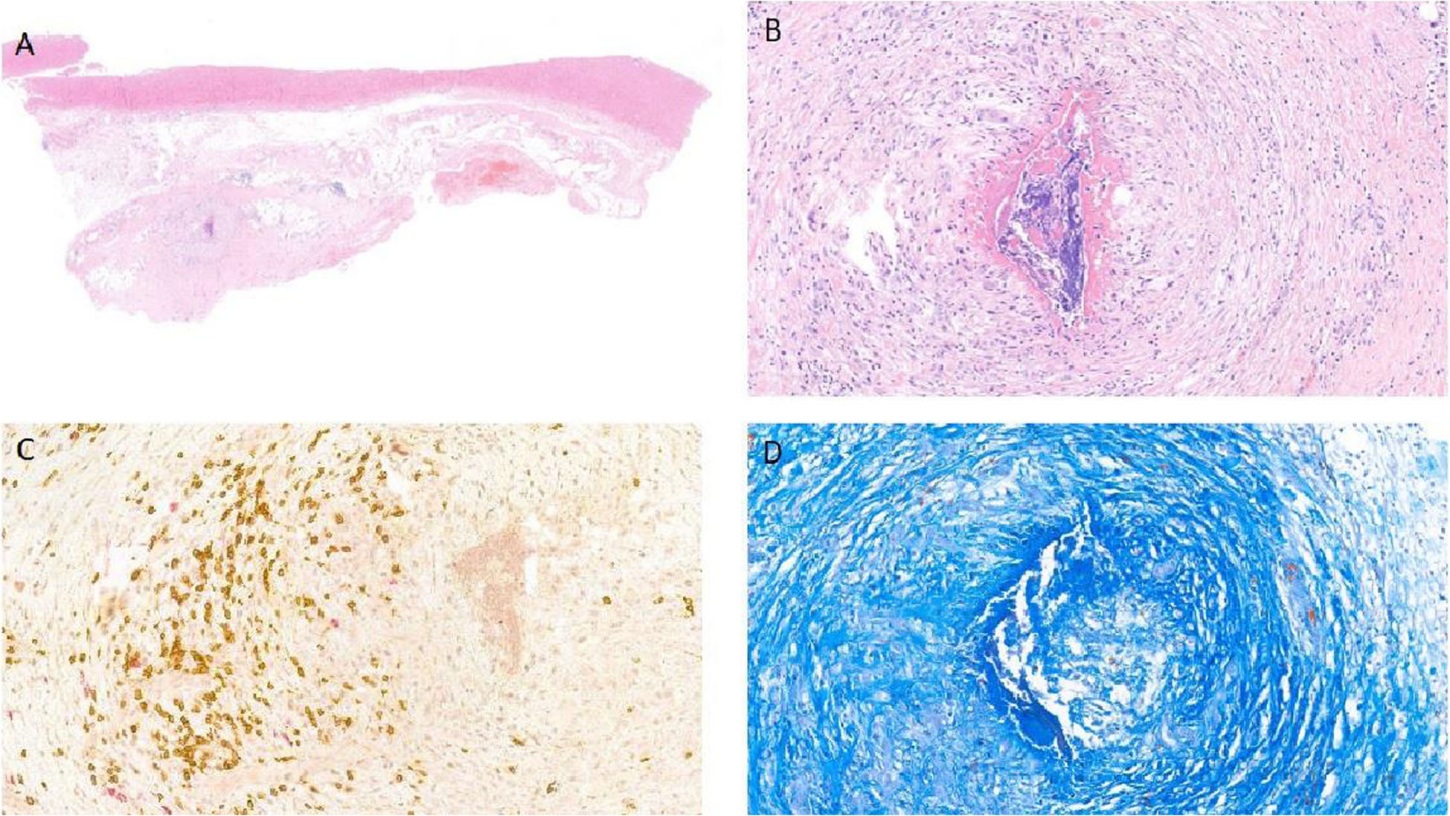
Histopathological section of the ascending aorta**:** Behçet’s disease involving the aorta. (**a**): Adventitial thickening is demonstrated using low-power magnification (HE, 10x) (**b**): Adventitial vasa vasoritis with luminal fibrotic narrowing (HE, 80x) and (**c**): moderate inflammatory infiltrate composed of mainly T-lymphocytes (brown), and small number of B-Lymphocytes (red) and macrophages (not labelled) in the wall (double staining immunohistochemistry, brown: CD3, red: CD20, 80x). (**d**): Concentric collagen deposition (dark blue) illustrating a chronic inflammation induced fibrosis (Crossmon, 80x)

After preoperative preparation in November 2019, the patient underwent an aortic root reconstruction with Bentall procedure using our modified conduit. Intraoperative findings confirmed the two pseudo-aneurysms. One pseudo-aneurysm originated between the non-coronary sinus and mitral annulus, while the other from behind the left main coronary branch. Furthermore, the prosthetic valve was fixed only by a shallow membrane protruding from the annulus. (Fig. 3.) As seen on CT angiography, intraoperatively we measured a dilated left ventricular outflow tract (LVOT) of 55 mm. We resected the prosthetic valve and the aortic root, and the coronary buttons were prepared. Both pseudo-aneurysm orifices were closed with a 4–0 polypropylene running suture. The proximal anastomosis was performed by suturing the skirt of the conduit directly to the myocardium of LVOT and to the mitral valve annulus with a 3–0 polypropylene running suture. (Fig. 4.) The distal anastomosis was covered with a collar made from the vascular graft in order to prevent the formation of a pseudo-aneurysm along the distal suture line. Cross-clamp time and ECC time were 156 min and 207 min, respectively. Complete atrioventricular block developed during surgery as the conduit was sutured to LVOT. Histological examination revealed adventitial thickening and adventitial perivascular infiltration dominated by lymphocytes and concentric collagen deposition illustrating a chronic inflammation-induced fibrosis. (Fig. 5.)

### Follow-up and outcomes

**Fig. 6:**
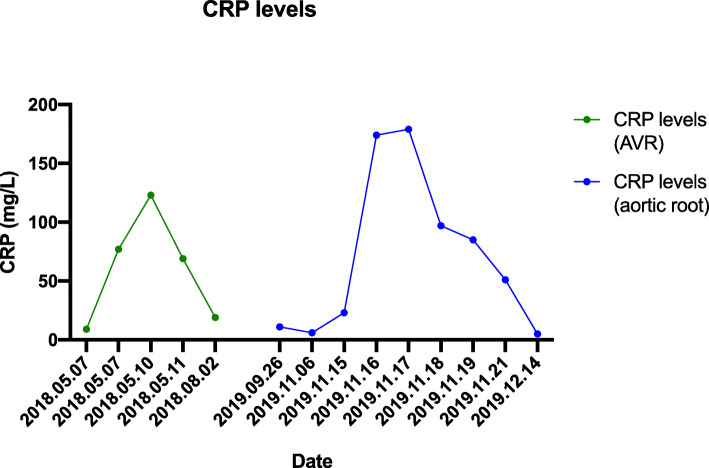
CRP level variations: The perioperative CRP concentration of primer aortic valve replacement is showed in green curve. The uneventful period is marked with grey dots. The perioperative CRP concentration of aortic root reconstruction is marked with blue curve. CRP concentrations were appropriate to perioperative period of cardiac surgery using ECC

After cardiac surgery, our patient underwent a permanent DDD pacemaker implantation (Medtronic Ensura DR; Medtronic PLC, Dublin, Ireland). Further postoperative course was uneventful. Postoperative echocardiography revealed moderately decreased left ventricular function with an ejection fraction of 44%. The prosthetic valve was properly functioning. Blood tests showed increased levels of inflammatory markers, but adequate to postoperative status (CRP 51 mg/l). One-month postoperative echocardiography confirmed a preserved left ventricular function with an ejection fraction of 55% and a normally functioning prosthetic valve. Chest CT scan showed a good postoperative result. In the proximate postoperative course corticosteroid immune therapy was applied, then escalated to adalimumab and methotrexate after 1 month after the operation. One-month CRP concentration was 5 mg/l. (Fig. 6.)

## Discussion and conclusions

### Discussion

Behçet’s disease is an auto-inflammatory disorder categorized as a primer systemic vasculitis. Recurrent symptoms occur as oral and genital ulcerations, uveitis, and erythema nodosum. Multisystemic manifestations occur, including arthritis, neurologic symptoms, gastrointestinal lesions, and cardiovascular complications. The aetiology is still unknown. Genetic factors such as the presence of the HLA-B51 gene, infectious agents and the irregularity of T-cell homeostasis are considered to be responsible for the emergence of Behçet’s disease. Pharmacological treatment of Behçet’s disease is based on the use of corticosteroid monotherapy or combined with other immunomodulatory drugs such as methotrexate, azathioprine, or anti-TNF-α antibodies.

Cardiovascular manifestations of BD affect nearly 30% of the patients. Aortic regurgitation seems to be a less frequent manifestation, though its outcome may be fatal. The surgical outcome of regular AVR is poor and the use of different surgical approaches is controversial. Prosthetic valve detachment occurs in 40–78% of cases after AVR [12]. Formation of pseudo-aneurysms of the aortic root or ascending aorta can also be present. Neutrophil infiltration is seen histologically, which results in weakened endothelial tissue. Reoperation is needed after a regular AVR in more than 50% of the cases [13].

In BD patients with severe aortic valve disease, the first choice of surgical technique should be aortic root reconstruction combined with immunomodulation therapy with low inflammatory levels. Aortic root reconstruction prevents prosthetic valve dehiscence and the formation of pseudo-aneurysms. After unsuccessful AVR operations, resection of the torn and weakened annulus and placement of the suture line directly to the left ventricular outflow tract can also be beneficial in well-selected cases.

### Conclussion

We presented a case of a middle-aged male patient with Behçet’s disease after AVR. Several months after the first operation he developed a severe paravalvular leak (PVL) and aortic root pseudo-aneurysm with an extremely dilated left ventricular outflow tract. Aortic root reconstruction was performed as reoperation. We created a special graft to handle the extreme left ventricular outflow tract dilatation. Our special conduit with a „skirt” is sizeable to the patient’s parameters and it might provide less tension between the suture lines of the conduit and the left ventricular outflow tract. The surgical outcome was satisfying in our case, as the 6-month follow up showed an excellent postoperative result.

## Data Availability

Not applicable.

## Abbreviations

AR: Aortic Regurgitation
AVR: Aortic Valve Replacement
BD: Behcet’s Disease
CRP: C Reactive Protein
ECC: Extracorporal Circulation
EDD: End Diastolic Diameter
ESD: End Systolic Diameter
HLA: Human Leukocyte Antigen
HSP: Heat Shock Protein
IL: Interleukin
LVOT: Left ventricular Outflow Tract
PVL: Paravalvular Leak
TNF: Tumor Necrosis Factor
